# Simple Adaptive Single Differential Coherence Detection of BPSK Signals in IEEE 802.15.4 Wireless Sensor Networks

**DOI:** 10.3390/s18010052

**Published:** 2017-12-26

**Authors:** Gaoyuan Zhang, Hong Wen, Longye Wang, Ping Xie, Liang Song, Jie Tang, Runfa Liao

**Affiliations:** 1 School of Electronic and Information Engineering, Henan University of Science and Technology, Luoyang 471023, China; xieping_1984@bupt.edu.cn (P.X.); l.song@utoronto.ca (L.S.); 2 National Key Laboratory of Science and Technology on Communications, University of Electronic Science and Technology of China, Chengdu 611731, China; sunlike@uestc.edu.cn (H.W.); 201311260116@std.uestc.edu.cn (L.W.); cs.tan@163.com (J.T.); liaorunfa_leo@foxmail.com (R.L.); 3 School of Engineering and Technology, Tibet University, Lhasa 850000, China

**Keywords:** single-symbol noncoherent detection, adaptive estimation, IEEE 802.15.4, wireless sensor networks

## Abstract

In this paper, we propose an adaptive single differential coherent detection (SDCD) scheme for the binary phase shift keying (BPSK) signals in IEEE 802.15.4 Wireless Sensor Networks (WSNs). In particular, the residual carrier frequency offset effect (CFOE) for differential detection is adaptively estimated, with only linear operation, according to the changing channel conditions. It was found that the carrier frequency offset (CFO) and chip signal-to-noise ratio (SNR) conditions do not need a priori knowledge. This partly benefits from that the combination of the trigonometric approximation $\sin^{-1}\left(x\right)\approx x$ and a useful assumption, namely, the asymptotic or high chip SNR, is considered for simplification of the full estimation scheme. Simulation results demonstrate that the proposed algorithm can achieve an accurate estimation and the detection performance can completely meet the requirement of the IEEE 802.15.4 standard, although with a little loss of reliability and robustness as compared with the conventional optimal single-symbol detector.

## 1. Introduction

Even since its introduction, the IEEE 802.15.4 low rate wireless personal area network (LR-WPAN) standard has found wide application in low complexity, low cost, low power consumption, and low data rate wireless connectivity among inexpensive devices [1], e.g., the smart sensor node in pervasive Wireless Sensor Networks (WSNs) [2]. WSNs can be applied to home automation, precision agriculture, health care, consumer electronic, industrial wireless control, environmental monitoring, and data collecting in battlefield awareness [3].

The specifications of the physical (PHY) layer and the medium access control (MAC) layer for IEEE 802.15.4 are defined in [1]. Much focus on its MAC protocol and capacity analysis has been witnessed in recent years [3,4,5]. Specially, Piyush et al. detailedly analyzed the capacity of WSNs, where *n* nodes are located in a region of area 1 m${}^{2}$. However, as we know, there is relatively less attention on the reliability issue in PHY layer, which is concerned in this paper. Specifically, the noncoherent signal detection algorithm for IEEE 802.15.4 WSNs is studied.

A binary phase shift keying (BPSK) direct sequence spread spectrum (DSSS) scheme is required in 868/915/950 MHz bands as indicated in [1]. A noncoherent receiver is preferred as compared to a coherent one in IEEE 802.15.4 BPSK receivers. This is because carrier acquisition and tracking is not required at the receiver in the battery-operated sensor node, although the complexity simplification is achieved at the cost of a compromise in detection performance. This degradation is often directly manifested in terms of the required signal-to-noise ratio (SNR) for a given bit error rate (BER) or packet-error rate (PER) [6,7].

Bit-level single differential coherent detection (SDCD) is known to be more popular in IEEE 802.15.4 BPSK receivers because of its attractive performance [2,8]. This follows that the chip-level SDCD suffers from serious performance loss due to chip-error propagation [9], while the bit-level SDCD combines soft decision values of the chip samples for detection and compensates for the constant carrier frequency offset effect (CFOE) in statistics after differential decoding (DD) [2].

To the best of our knowledge, all available detectors classified into this category [2,8,10] employ a two-step approach referred to here as the bit-level SDCD (BSDCD) approach: Step 1 (postcompensation step) estimates the residual CFOE based on a measurement derived from the whole preamble samples at the autocorrelator output and then feeds back a coherent reference to the following autocorrelation sample of the useful data; and Step 2 (decision step) detects the data bit-by-bit. The idea in the first step seems to be economically attractive for WSNs because of its simple implementation. Furthermore, the  second step combines the simplicity of the approach without any compensation, with the improved performance due to including some degree of simple phase rotation of the detection statistic after DD, i.e., the differential detector output. Hence, this unified *estimation*-*detection* framework appears to offer a very reasonable trade-off between error performance and detection complexity.

This unified heuristic configuration can be considered an inspiration from the idea of the Generalized Likelihood Ratio Test (GLRT) [11], yet the receiver has partial prior knowledge of the nuisance parameter (e.g., an unknown CFOE) from known symbols (e.g., the preamble symbols). In principle, it can be interpreted as an approximation to the optimal coherent detectors [12]. The creative approximation is imperative for IEEE 802.15.4 WSNs where computation of the optimal detector is too difficult and uneconomical. It can also be classified into the data-aided (DA) noncoherent demodulation [13].

It is important to note that a similar approach was also introduced by Liu et al. in [14] for *M*-ary phase shift keying (*M*-PSK) with single differential (SD) coding. However, in this case, the CFO embedded in samples at differential detector input is considered to be recovered from some unknown modulated symbols. Therefore, the nonlinear operation (e.g., *M*th power for *M*-PSK) [15] is required to eliminate the dependence of these datas as indicated in Equation (7) of [14]. This is because the undesired parameter CFO is now a parameter of interest, whereas the data symbols are now just nuisance parameters.

For the aforementioned postcompensation step, several CFOE estimation approaches have been studied [2,8,10]. The full estimation scheme is powerful yet always not affordable, since the inverse tangent operation is considered to be the most complex to implement in WSNs [2]. The work of Lee et al. [8] first successfully simplifies this implementation; however, a considerable performance compromise cannot be avoided. This is because the mathematical approximation $\tan^{-1}\left(x\right)\approx 0$ is considered therein and thus the CFOE is quantified into four fixed values (in radians), i.e., 0,π/2,−π, and −π/2 [10]. These phases clearly seems to be independent of the channel conditions, i.e., SNRs. Considering the more advisable trigonometric approximation $\tan^{-1}\left(x\right)\approx x$, we recently introduced an adaptive offset-term adjustment algorithm in [10]. It has the advantage of enabling the receiver to intelligently adjust the four constant phases above according to the time-varying channel conditions with respect to each packet transmission. However, a division operation is required to perform this process. This needs a complex implementation and is energy-consuming in floating-point form [16]. An efficient symbol-timing algorithm was also deeply discussed by Bloch et al. in [2], where the observation is necessary to be extended to three bit intervals. In this scheme, the most recent bit detection result is required in practice as shown in Equation (8) of [2]. Under this decision-feedback mechanism, error propagation may take place. For more details about these eatimators, the reader is referred to [10] and the reference therein.

The main contribution of this work can be highlighted as follows. The above so-called BSDCD technique is first deduced in detail, and its receiver structure is given. Then, for the compensation processor, some particular estimators are summarized and compared. Finally, an efficient estimator with only addition operation is provided for the compensation processor. It is worth noting that the high-order modulation [17], e.g., offset quadrature phase-shift keying (O-QPSK) with half-sine pulse shape, is also employed in IEEE 802.15.4 WSNs [1]. A simple double correlation based detector has been derived in [18,19], which however is out of the scope of this work.

To the best of our knowledge, this is the first work investigating the combination of the trigonometric approximation $\sin^{-1}\left(x\right)\approx x$ and the high chip SNR to simplify the optimal detector. The remainder of this paper is organized as follows. Section 2 concentrates on the signal model over an additive white Gaussian noise (AWGN) channel. The generalized BSDCD algorithm is explicitly proposed in Section 3, while the optimal BSDCD scheme is described in Section 4. Section 5 indicates several particular CFOE estimation schemes for the postcompensation process, and a new estimator is proposed in Section 6. Numerical results are addressed in Section 7. Finally, some conclusions and future work are offered in Section 8.

## 2. System Model

Consider transmission over an AWGN channel and ideal timing synchronisation at the receiver in sensor node. The CFO and carrier phase offset (CPO) are assumed to be unknown and random but constant with respect to a packet transmission [2]. Without loss of generality, we follow the discrete-time signal model in [2] but with some changes. In particular, the baseband equivalent chip sequence for the *m*th bit E[m] is

(1) $$\begin{array}{c} r_{m,k}=s_{m,k}e^{j(2\pi kfT_{c}+\theta )}+\eta_{m,k},\;1\leq k\leq K. \end{array}$$

Here, $s_{m,k}$ is the *k*th bipolar BPSK modulated chip, *f* is the CFO in Hz, and θ is arbitrary modulo-2π reduced CPO in radians. $T_{c}$ is the chip duration, $\eta_{m,k}$ is a discrete-time, circularly symmetric, zero-mean complex AWGN with variance $\sigma_{m,k}^{2}$, and K=15 is the length of the pseudorandom number (PN) code [1]. We assume that *f* and θ are statistically independent of each other, and of the AWGN $\left\{\eta_{m,k}\right\}$.

Notice that the channel is supposed to only introduce CPO, but otherwise is assumed to be perfectly equalized. That is to say, a phase noncoherent channel is considered. Signal distortion caused by multipath fading is not considered for simplicity in describing detection principle, i.e., the indoor short-range propagation model is used, where the complex channel gain equals to 1 [19]. The CFO is due to instabilities associated with the inexpensive carrier oscillators in transmitting node and receiving node. The bandwidth of the receiving filter *B* is wide enough for all of the signal energy spread by CPO and CFO to pass [20]. In this case, all of the modeling and analysis in this work can be performed using the discrete-time complex baseband model in [8].

## 3. Generalized BSDCD Scheme

The receiver performs BSDCD of the *m*th bit E[m] by forming a complex-represented statistic from sample autocorrelation operation [8]:

(2) $$\begin{array}{c} A\left[m\right]=\sum_{k=1}^{L_{1}}r_{m,k}r_{m-1,k}^{*}=L_{1}e^{j\phi_{m}}e^{jK\omega T_{c}}+\eta \left(0\right), \end{array}$$

where $L_{1}$ is the sample number, $1\leq L_{1}\leq K$, the superscript ∗ is complex conjugate, $\phi_{m}\in \left\{0,\pi \right\}$ is the actual information phase corresponding to the *m*th transmitted bit E[m], ω=2πf, and η(0) is an integrated noise sample.

Obviously, first-order differential modulation is a means of handling only one unknown invariable parameter in two adjacent bit intervals [14]. Thus, the CFOE $\varphi ≜K\omega T_{c}$ embedded in autocorrelator output A[m] is a residual nuisance parameter, generating a multiplicative phase distortion $e^{j\varphi }$ as indicated in Equation (2). It is by no means evident that this impairment, referred to as phase noise, can cause a significant performance loss if it is not wiped out. The avenue considered here is to equip the autocorrelator with a postcompensation processor. It not only is simple to implement but has the potential to be almost without performance degradation as the frequency shift increases. It is important to note that the exponential term $e^{jK\omega T_{c}}$ is referred as the CFOE in [2], but here is $K\omega T_{c}$.

Specifically, the CFOE is first recovered based on averaged autocorrelation samples of the preamble [8]:

(3) $$\begin{array}{c} Y=\frac{1}{\left(L_{2}-1\right)L_{3}}\sum_{m=2}^{L_{2}}\sum_{k=1}^{L_{3}}r_{m,k}r_{m-1,k}^{*}=e^{jK\omega T_{c}}+\eta \left(1\right). \end{array}$$

Here, $L_{2}$ is the observation length in bit intervals, and $2\leq L_{2}\leq J$, where J=32 is the bit number in preamble field [1]. $L_{3}$ is referred to as the number of the autocorrelation sample, $1\leq L_{3}\leq K$, and η(1) is another complex-valued noise term.

Then, follow with the result reported in Equation (18) of [6], a decision rule can be intuitively written as:

(4) $$\begin{array}{c} {\hat{\phi }}_{m}=\underset{\phi_{i}}{\arg\max}\;\Xi \left(A\left[m\right],\phi_{i},Y\right), \end{array}$$

where “argmax” denotes the argument that maximizes the following function with respect to the variable of interest, i.e., $\phi_{i}$. Here, $\phi_{i}\in \left\{0,\pi \right\}$ is the hypothetical information phase and $\Xi \left(A\left[m\right],\phi_{i},Y\right)$ denotes the detection metric, given by [6]

(5) $$\begin{array}{c} \Xi \left(A\left[m\right],\phi_{i},Y\right)=Re\left\{A\left[m\right]q\left(Y\right)e^{-j\phi_{i}}\right\}. \end{array}$$

In Equation (5), q(Y) is the so-called quantization function [2], which can be considered as a phase coherent reference to compensate the effect of the CFO on A[m]. Re{x} denotes the real part of *x*. Clearly, a bit detection error is generated when ${\hat{\phi }}_{m}\neq \phi_{m}$.

Observe from Equations (2) and (5) that the quantization function q(Y) is used to undo rotation induced by CFOE in autocorrelator output A[m]. Bearing in mind that the BPSK alphabet is {+1,−1}, we can immediately arrive at another detection rule from Equation (4) in the following form:

(6) $$\begin{array}{c} \hat{E}\left[m\right]=\left\{\begin{array}{c} 0,\;\mathrm{if}\;Re\{A[m]q(Y)\}\geq 0,\\ 1,\;\mathrm{if}\;Re\{A[m]q(Y)\}<0. \end{array}\right. \end{array}$$

This clearly is the receiver strategy followed in [2,8,10]. In fact, equip Equation (4) with respective quantization functions, and let truncationfactors $L_{1}=L_{3}=K$, $L_{2}=J$; then, we can arrive at the conventional detection approaches.

The details of Generalized BSDCD (GBSDCD) are presented in Algorithm 1. Note that the bits of the start-of-frame delimiter (SFD) in the first field and PHY header (PHR) in the second field of the physical layers protocol data unit (PPDU) are not considered in Algorithm 1. This simplicity is considered to make the ideas behind the detection principle apparent. In addition, the preamble field of the PPDU is composed of 32 binary zeros [1], therefore the transmitted bits E[m]=0, for 1≤m≤32. It is just based on these known bits that the postcompensation process can be performed at the receiver. This prior preamble field is primitively used for automatic gain control (AGC) convergence, diversity selection, timing acquisition, and coarse frequency acquisition in the IEEE 802.15.4 receivers [1,2].

The detector given here falls into those that use ad hoc methods [13], such as the decision-feedback-based method [21], the frequency-offset estimation-based method in [14] and the postcompensation-based method in this work. These algorithms can provide a wide range of trade-offs among detection complexity, detection speed, and PER performance.

**Algorithm 1** Framework of GBSDCD Algorithm.**Input:**
$r_{m,k}$: baseband sample for the *m*th bit E[m]; $L_{1}$: sample number for each bit of the actual data; $L_{2}$: observation length of the preamble in bit intervals; $L_{3}$: number of the autocorrelation sample *m*th bit of the preamble; *J*: bit number in preamble field; *L*: payload length of the PPDU. **Output:**
$\hat{E}\left[m\right]$: detection of the *m*th bit of the actual data.
1:initial Y=0, A[m]=0, and J=322:**for** m=2; $m\leq L_{2}$; m++**do**3: **for** k=1; $k\leq L_{3}$; k++ **do**4:  $A\left[m\right]\leftarrow A\left[m\right]+r_{m,k}r_{m-1,k}^{*}$5: **end for**6: Y←Y+A[m]7:**end for**8:compute the quantization function q(Y);9:**for** m=J+2; m≤J+L; m++**do**10: **for** k=1; $k\leq L_{1}$; k++ **do**11:  $A\left[m\right]\leftarrow A\left[m\right]+r_{m,k}r_{m-1,k}^{*}$12: **end for**13: Ξ←A[m]q(Y)14: **if** Ξ<0 **then**15:  $\hat{E}\left[m\right]\leftarrow 1$16: **else**17:  $\hat{E}\left[m\right]\leftarrow 0$18: **end if**19:**end for**20:**return** $\hat{E}\left[m\right]$


The role of the differential coding is to facilitate CPO estimation and effectively translate the noncoherent channel to a coherent AWGN channel. Furthermore, the role of the preamble symbols is to facilitate CFOE estimation and effectively wipe out the instabilities associated with the inexpensive carrier oscillators in transmitting node. It is such exact knowledge of these symbols that reduces the degree of randomness and facilitates the estimation of the undesired parameter φ of interest. Note that, in the postcompensation process, the undesired parameter φ is now the parameter of interest that we would like to estimate, whereas the “parameters” of interest in the detection process, namely the data symbols, are now just nuisance (or undesired) parameters (cf. [22], p. 64).

## 4. The Available Optimal Quantization Function

As described in the preceding section, the quantization function q(Y) can be considered as an estimator of the phasor $e^{-j\varphi }$ to eliminate the dependence of the residual nuisance parameter φ in autocorrelator output A[m]. It exhibits an important role not only in detection performance but in robustness to the CFO. Thus, in this section, we turn our attention towards developing q(Y) to achieve a benchmark that can be theoretically achieved.

In this case, q(Y) in Equation (5) can be expressed as:

(7) $$\begin{array}{c} q\left(Y\right)=Y^{*}=\left|Y\right|e^{-j∠Y}. \end{array}$$

Here, ∠Y is the principal value of the argument of the measurement *Y*, and |Y| denotes the magnitude of *Y*. The positive nature of magnitude |Y| means it does not affect the decision result in Equation (4). Hence, Equation (7) can be simplified as:

(8) $$\begin{array}{c} q\left(Y\right)=e^{-j\hat{\varphi }}, \end{array}$$

where $\hat{\varphi }$ must be set equal to the principal value of the argument of the measurement *Y*, i.e.,

(9) $$\begin{array}{c} \hat{\varphi }=\left\{\begin{array}{c} \tan^{-1}\left(\frac{Im(Y)}{Re(Y)}\right),\;\mathrm{if}\;Re\left(Y\right)>0,\\ \frac{\pi }{2},\;\mathrm{if}\;Re\left(Y\right)=0,Im\left(Y\right)\geq 0,\\ -\pi +\tan^{-1}\left(\frac{Im(Y)}{Re(Y)}\right),\;\mathrm{if}\;Re\left(Y\right)<0,\\ -\frac{\pi }{2},\;\mathrm{if}\;Re\left(Y\right)=0,Im\left(Y\right)<0. \end{array}\right.≜{\hat{\varphi }}_{1},\;\; \end{array}$$

Here, Im(Y) denotes the imaginary part of *Y*. Equation (9) is referred to here as the first old estimation model, and the receiver with it is the well-known conventional optimal receiver [2].

**Figure 1:**
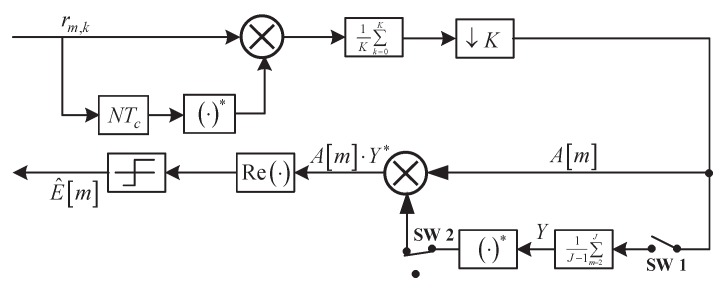
The optimal receiver structure.

The noise term η(1) in Equation (3) is not Gaussian but can be approximated as Gaussian when chip signal-to-noise ratio (SNR) is reasonably high [23]. As a consequence, we can think of the measurement *Y* in Equation (3) as the equivalent received signal at time *m* and η(1) as an equivalent AWGN channel. In such condition, Equation (9) can be interpreted as data-aided maximum likelihood (DAML) estimates of the phase φ and Equation (8) a DAML estimate of the phasor $e^{-j\varphi }$ (cf. [24], p. 166). In this case, the receiver structure is given in Figure 1 [25]. In Figure 1, the switch 1 (SW 1) should open in the bit intervals 1≤m≤32, and be off otherwise. For SW 2, the opposite occurs.

We observe immediately from Equation (9) that the arctan operation cannot be avoided in the postcompensation process of the optimal receiver. This requires a computational complexity that renders its theoretical approach impractical in WSNs, where low power and low cost are of paramount importance. Therefore, the creative approximation, thus avoiding intractable complexity in this process, is imperative for IEEE 802.15.4 WSNs where computation of the optimal detector is too difficult and uneconomical.

## 5. Summary and Analysis of Some Simplified Estimation Schemes

### 5.1. Simplified Estimation—Estimation A

Assuming that small CFO and high chip SNR remain $\left|\frac{Im(Y)}{Re(Y)}\right|$ sufficiently small, we can use the simple approximation that $\tan^{-1}\left(x\right)\approx x$, for small *x*, and Equation (9) can be directly approximated as

(10) $$\begin{array}{c} \hat{\varphi }\approx \left\{\begin{array}{c} \frac{Im(Y)}{Re(Y)},\;\mathrm{if}\;Re\left(Y\right)>0,\\ \frac{\pi }{2},\;\mathrm{if}\;Re\left(Y\right)=0,Im\left(Y\right)\geq 0,\\ -\pi +\frac{Im(Y)}{Re(Y)},\;\mathrm{if}\;Re\left(Y\right)<0,\\ -\frac{\pi }{2},\;\mathrm{if}\;Re\left(Y\right)=0,Im\left(Y\right)<0. \end{array}\right.≜{\hat{\varphi }}_{2},\;\; \end{array}$$

The detecor with Equation (10) works only for small CFO and reasonably high SNRs. This is because $\left|\frac{Im(Y)}{Re(Y)}\right|\leq 1$ is guaranteed with very high probability in this condition, and the detection effect of approximate error in $\tan^{-1}\left(x\right)\approx x$ will be as small as we expect. Otherwise, large mathematical approximation error will introduce excessive estimation error, and, finally, unacceptable detection penalty cannot be avoided.

### 5.2. Simplified Estimation—Estimation B

In order to obtain a low-complexity estimator that is not only as efficient as Equation (9) but valid for any cases, an equivalent description for Equation (9) was given in [10] by

(11) $$\begin{array}{c} \hat{\varphi }=\left\{\begin{array}{c} \;\tan^{-1}\left(\frac{Im(Y)}{Re(Y)}\right),\;\mathrm{if}\;Re\left(Y\right)>0\;\mathrm{and}\;|Re\left(Y\right)|\geq |Im\left(Y\right)|,\\ \;\frac{\pi }{2}-\tan^{-1}\left(\frac{Re(Y)}{Im(Y)}\right),\;\mathrm{if}\;Im\left(Y\right)>0\;\mathrm{and}\;|Re\left(Y\right)|<|Im\left(Y\right)|,\\ -\pi +\tan^{-1}\left(\frac{Im(Y)}{Re(Y)}\right),\;\mathrm{if}\;Re\left(Y\right)<0\;\mathrm{and}\;|Re\left(Y\right)|\geq |Im\left(Y\right)|,\\ -\frac{\pi }{2}-\tan^{-1}\left(\frac{Re(Y)}{Im(Y)}\right),\;\mathrm{if}\;Im\left(Y\right)<0\;\mathrm{and}\;|Re\left(Y\right)|<|Im\left(Y\right)|. \end{array}\right.\;\;\;\;\; \end{array}$$

Equations (9) and (11), originally obtained using different intuitive reasonings, are two approaches for computing the principal value of the argument of the measurement *Y*.

**Figure 2:**
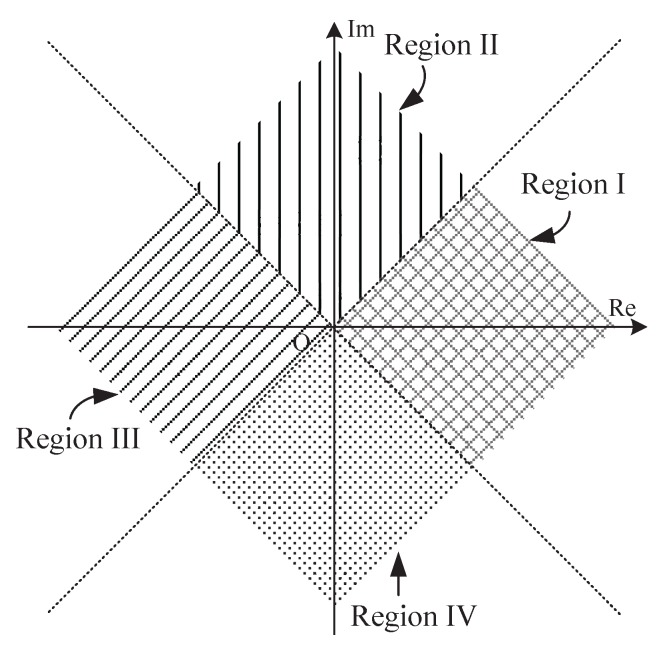
Division of the complex observation space.

We observe immediately from Equation (11) that the complex observation space is subdivided into four equi-angular sectors illustrated in Figure 2, which are identical to the four regions presented in Table 1 of [8]. Both of them can be distinguished from one another by only simple comparison of the measurement magnitudes and signs of real and imaginary parts of *Y*. From this viewpoint, Equation (11) differs from Equation (9) in two respects: (1) the observation space is further subdivided into four subspaces, not two; and (2) the subspace locating criteria is redesigned. It is such characteristics that make Equation (11) attractive for devising a simplified estimator as shown in the following.

As indicated in Equation (11), $\left|\frac{Im(Y)}{Re(Y)}\right|$ or $\left|\frac{Re(Y)}{Im(Y)}\right|$ in an observation region is not only never more than 1, but irrelevant to CFOE being estimated or SNR conditions. This appropriately allows us to use the mathematical approximation that $\tan^{-1}\left(x\right)\approx x$, for small *x*, in Equation (11) without undesirable calculation errors. Then, another efficient simplified detection scheme was proposed in our recent work [10], where the estimator is valid for arbitrary CFO and SNR, given by

(12) $$\begin{array}{c} \hat{\varphi }\approx \left\{\begin{array}{c} \frac{Im(Y)}{Re(Y)},\;\mathrm{if}\;Re\left(Y\right)>0\;\mathrm{and}\;|Re\left(Y\right)|\geq |Im\left(Y\right)|,\\ \frac{\pi }{2}-\frac{Re(Y)}{Im(Y)},\;\mathrm{if}\;Im\left(Y\right)>0\;\mathrm{and}\;|Re\left(Y\right)|<|Im\left(Y\right)|,\\ -\pi +\frac{Im(Y)}{Re(Y)},\;\mathrm{if}\;Re\left(Y\right)<0\;\mathrm{and}\;|Re\left(Y\right)|\geq |Im\left(Y\right)|,\\ -\frac{\pi }{2}-\frac{Re(Y)}{Im(Y)},\;\mathrm{if}\;Im\left(Y\right)<0\;\mathrm{and}\;|Re\left(Y\right)|<|Im\left(Y\right)|, \end{array}\right.≜{\hat{\varphi }}_{3}.\;\; \end{array}$$

### 5.3. Simplified Estimation—Estimation C

A complexity efficient detector was also deeply considered in [8]. Actually, *Y* is quantized into 1, −1, *j* and −j in this detector, a result later used in [2]. It follows from this that its estimator can be depicted by [10]

(13) $$\begin{array}{c} \hat{\varphi }\approx \left\{\begin{array}{c} 0,\;\mathrm{if}\;Re(Y)>0\;\mathrm{and}\;|Re(Y)|\geq |Im(Y)|,\\ \frac{\pi }{2},\;\mathrm{if}\;Im\left(Y\right)>0\;\mathrm{and}\;|Re\left(Y\right)|<|Im\left(Y\right)|,\\ -\pi ,\;\mathrm{if}\;Re(Y)<0\;\mathrm{and}\;|Re(Y)|\geq |Im(Y)|,\\ -\frac{\pi }{2},\;\mathrm{if}\;Im\left(Y\right)<0\;\mathrm{and}\;|Re\left(Y\right)|<|Im\left(Y\right)|, \end{array}\right.≜{\hat{\varphi }}_{4}.\;\; \end{array}$$

Obviously, Equation (13) can be derived from Equation (11) on condition that the approximation $\tan^{-1}\left(x\right)\approx 0$ is involved. This avenue achieves an estimator with perfectly acceptable complexity. The detector with it works only for the CFOE approach to four fixed values, i.e., 0, π/2, −π and −π/2; otherwise, a large error similar to Equation (10) will be introduced. Not surprisingly, this is expected to limit the receiver performance. A quick comparison of Equations (12) and (13) reveals that the estimation error in the latter can be corrected by the former, where two types of adaptive offset terms $\frac{Im(Y)}{Re(Y)}$ and $\frac{Re(Y)}{Im(Y)}$ are additionally provided to improve the estimated accuracy of the time-variant undesired parameter φ.

### 5.4. Remarks

Some comments on the above estimators are as follows:

- These estimators are obtained from approximations made to a structure motivated by DAML estimation of the CPOE.
- The estimator in Equation (10) is convenient and with acceptable complexity as compared with Equation (9). Since it works only for very small frequency-offset values and reasonably high SNRs, it is a CFO and SNR limited estimator. Correspondingly, a severe degradation to the system packet error performance in the detection process will be introduced, which is not suitable for our purposes.
- Equation (12) achieves an estimate with quite reasonable accuracy. It does so by four preconditions, i.e., smart geometric division to the observation space, accurate subspace locating criterion, equivalent avenue for the calculation of ∠Y, and advisable approximation $\tan^{-1}\left(x\right)\approx x$. One arctan operation is required for a DAML estimator given in Equation (9). However, this computationally intensive operation is completely avoided in Equation (12). Surprisingly, such a complexity reduction results in almost no frequency-offset invariance degradation in the detection process, as will be inferred in Section 7.
- The canonical approximation $\tan^{-1}\left(x\right)\approx x$ involved in Equation (12) is much more accurate than the atypical approximation $\tan^{-1}\left(x\right)\approx 0$ involved in Equation (13). This implies that, as such inaccuracy frequently makes the measurement in Equation (13), either a large overestimation or a large underestimation of the CFOE, whereas no such inappropriate estimation is likely to occur in Equation (12). Actually, Equation (12) allows a real-time adjustment with additional one division and one addition operations, which, however, can achieve almost 1.5 dB gains at PER of $1\times 10^{-3}$ [10].

In a word, Equation (12) makes a detector with such a reasonable balance between complexity of the postcompensation step and performance of the detection step that it may be more attractive for WSNs. However, a division operation is still required as indicated in Equation (12). Clearly, division is an energy intensive and costly operation [16], and should be avoided as much as possible in WSNs. In the following, we are concerned with further simplication to wipe off this complex nonlinear operation in the first processing, while without unaccepted performance degradation in the second processing.

## 6. Proposed Divisor-Free CFOE Estimator

The starting point of our estimation approach is the realization that the modulo-2π reduced phase of the measurement *Y* can also be expressed in form of $\sin^{-1}\left(x\right)$, i.e., Equation (9) can be rewrote as follows:

(14) $$\begin{array}{c} ∠Y=\left\{\begin{array}{c} \;\sin^{-1}\left(\frac{Im(Y)}{\left|Y\right|}\right),\;\mathrm{if}\;Re\left(Y\right)\geq 0\\ -\pi -\sin^{-1}\left(\frac{Im(Y)}{\left|Y\right|}\right),\;\mathrm{if}\;Re\left(Y\right)<0 \end{array}\right..\;\; \end{array}$$

Observe from Equation (14) that the absolute value of the magnitude divided argument of the inverse sine function is never more than 1 because length of any right-angle side is never more than that of the hypotenuse. Then, we can immediately simplify Equation (14) as follows:

(15) $$\begin{array}{c} ∠Y\approx \left\{\begin{array}{c} \;\frac{Im(Y)}{\left|Y\right|},\;\mathrm{if}\;Re\left(Y\right)\geq 0,\\ -\pi -\frac{Im(Y)}{\left|Y\right|},\;\mathrm{if}\;Re\left(Y\right)<0. \end{array}\right.\;\; \end{array}$$

Note that the mathematical approximation $\sin^{-1}\left(x\right)\approx x$, for small *x*, is involved in Equation (15).

Assuming further that high SNR, known to be useful in estimation problems [26], keeps the magnitude of the non-Gaussian noise term η(1) in Equation (3) sufficiently small, we get

(16) $$\begin{array}{c} \left|Y|=\right|e^{jK\omega T_{c}}+\eta \left(1\right)|\approx 1. \end{array}$$

It is worth noting that this simplification is irrespective of the frequency offset being estimated. Substituting Equation (16) into (15), we can finally improve a CFOE estimator as follows:

(17) $$\begin{array}{c} {\hat{\varphi }}_{new}≜\left\{\begin{array}{c} \;Im(Y),\;\mathrm{if}\;Re(Y)\geq 0,\\ -\pi -Im(Y),\;\mathrm{if}\;Re(Y)<0. \end{array}\right.\;\; \end{array}$$

Note that Equation (14) can be rewritten in the following form:

(18) $$\begin{array}{c} ∠Y=\left\{\begin{array}{c} \;\sin^{-1}\left(\frac{Im(Y)}{\left|Y\right|}\right),\;\mathrm{if}\;Re\left(Y\right)\geq 0,\\ \pi -\sin^{-1}\left(\frac{Im(Y)}{\left|Y\right|}\right),\;\mathrm{if}\;Re\left(Y\right)<0. \end{array}\right.\;\; \end{array}$$

Then, Equation (17) can also be given by:

(19) $$\begin{array}{c} {\hat{\varphi }}_{new}≜\left\{\begin{array}{c} \;Im(Y),\;\mathrm{if}\;Re(Y)\geq 0,\\ \pi -Im(Y),\;\mathrm{if}\;Re(Y)<0. \end{array}\right.\;\; \end{array}$$

We provide some interpretations for the receiver whose estimator is expressed by Equation (19).

**Figure 3:**
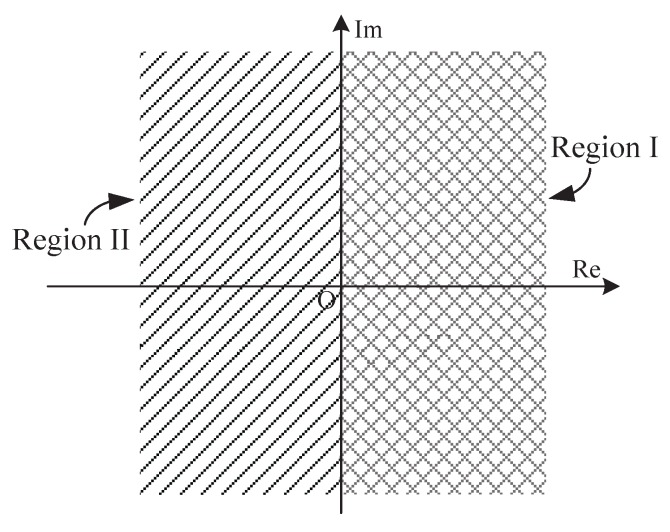
New division of the complex observation space.

**Figure 4:**
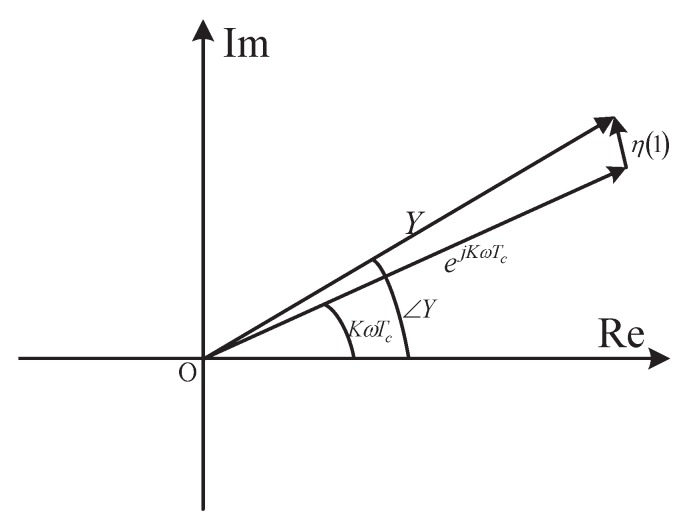
Geomrtirc representation of the measurement *Y*.

**Figure 5:**
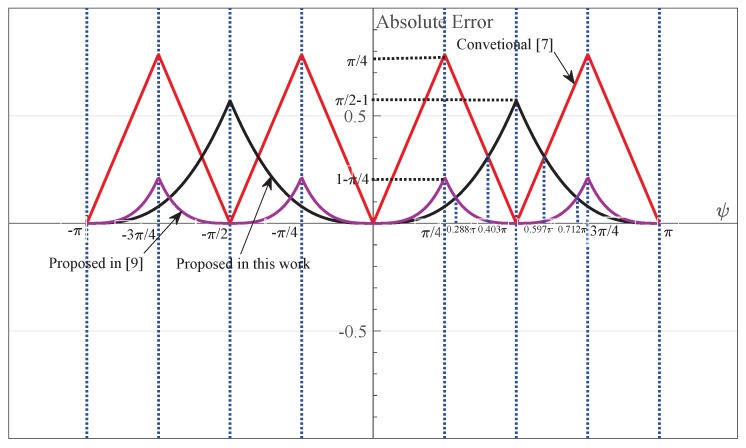
The absolute error |e| of three different estimation methods.

- Equation (19) are obtained from approximations made to a structure motivated by DAML estimation scheme in Equation (14) or Equation (18), and a simple adaptive offset terms Im(Y) is provided. This enables the receiver to intelligently adjust the estimated CPOE value according to the time-varying channel conditions with respect to each packet transmission.
- The complex observation space is only divided into two subspaces as shown in Figure 3. They can be easily distinguished from one another just with the sign of the real part of *Y*. Thus, the absolute value operation of real and imaginary parts of *Y* in Equation (12) is not required in this case. An useful assumption, namely, the asymptotic or high chip SNR, is considered for simplification of Equation (15). Thus, the nonlinear division operation in Equation (12) is avoided.
- No limitation on the CFOE range is required for the mathematical approximation $\sin^{-1}\left(x\right)\approx x$ in our estimator. That is to say, it appears to be a full-range estimator, and the corresponding receiver is a full-range detector, the same feature as the receiver with Equation (12). By the approximation |Y|≈1 valid at high chip SNR as illustrated in Figure 4, the result in Equation (19) may be shown to approximately hold for arbitrary SNRs, which is also the same as Equation (12). In a word, the CFO and chip SNR conditions do not need a priori knowledge for the mathematical approximation involved in Equation (19). As a result, an excellent detection performance is achieved at the receiver, which can completely meet the requirements of the IEEE 802.15.4 standard. This will be confirmed by the numerical results shown in the following section.
- Clearly, the estimation error $e≜\hat{\varphi }-\varphi$ is only introduced by the mathematical approximation if the noise is not considered. In this case, the absolute error |e| for three simplified estimators is summarized and compared in Table 1 and Figure 5, respectively. As shown in Figure 5, the absolute error |e| with Equation (12) is no more than 1−π/4 for which the observation space is divided into four subspaces as indicated in Figure 2. It increases to π/4 for Equation (13). This is exactly half of the radians for the equi-angular sector in Figure 2. This value comes to be π/2−1 for our improved estimator in Equation (19) where the observation space is only divided into two subspaces. Furthermore, when the CFOE φ∈(−π,−0.712π)∪(−0.288π,0.288π)∪(0.712π,π), our estimator is more efficient than Equation (12).

## 7. Numerical Results and Discussion

In this section, we evaluate the BER and PER performances of some BSDCD schemes. In all simulations, the payload length of the physical layer protocol data unit (PPDU) is 20 octets [1], and the detection procedure is repeated until enough error packets are collected. The carrier frequency was selected as 924 MHz, i.e., the maximum values required in a 915 MHz band [1]. All of these parameters are described in detail in Table 2. Note that the bits in the PHR, which indicates the number of octets of the PPDU, are also considered in all simulations. All experiments in this section were developed on a personal computer (3.2 GHz, 8 GB RAM) in a MATLAB (R2017a) (MathWorks, Beijing, China) platform.

### 7.1. Effect of the Truncation Factors

**Figure 6:**
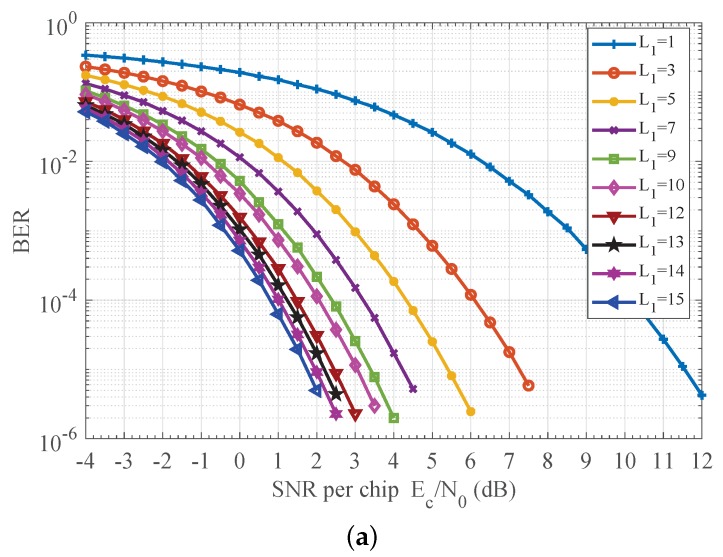

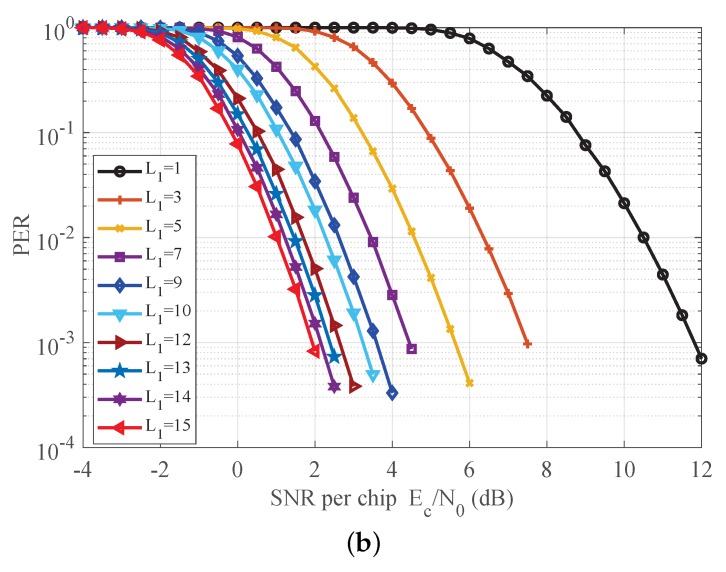
Performance impact of parameter $L_{1}$. (**a**) BER impact of $L_{1}$; (**b**) PER impact of $L_{1}$.

The truncation factors $L_{1}$, $L_{2}$ and $L_{3}$ regulate the complexity and performance, so we first give evaluation results in Figure 6 for the proposed detector, which provides us with the effect $L_{1}$ has on detection performance. Here, the CFO was randomly set to range from −80 ppm to 80 ppm with probability density function of symmetric triangular shape [2]. The CPO was considered to be uniform distribution in interval (−π, π). We observe that performance improves as sample length $L_{1}$ increases from 1 to 15, but improvement degrades as sample length $L_{1}$ increases. For example, Figure 6 shows that for PER of $1\times 10^{-3}$, as sample length $L_{1}$ increases from 1 to 3, the SNR gain is about 4.3 dB. As $L_{1}$ increases from 5 to 7, the SNR gain reduces to 1.2 dB. As $L_{1}$ further increases from 13 to 15, the SNR gain becomes 0.5 dB. Similar results were also achieved for $L_{2}$ and $L_{3}$, which are not given here. In pratice, suitable truncation factors can be selected according to the specific requirements on the performance, and a large degree of freedom in complexity can be achieved. For example, according to [1], the PER should be less than $1\times 10^{-2}$ when the SNR is 5 to 6 dB. Referring to Figure 6, five samples are sufficient when the SNR of the input chip signal is 5 dB. In the following, the maximum values are considered for $L_{1}$, $L_{2}$ and $L_{3}$ to ensure the best possible performance.

### 7.2. Detection Performance of the Receiver

**Figure 7:**
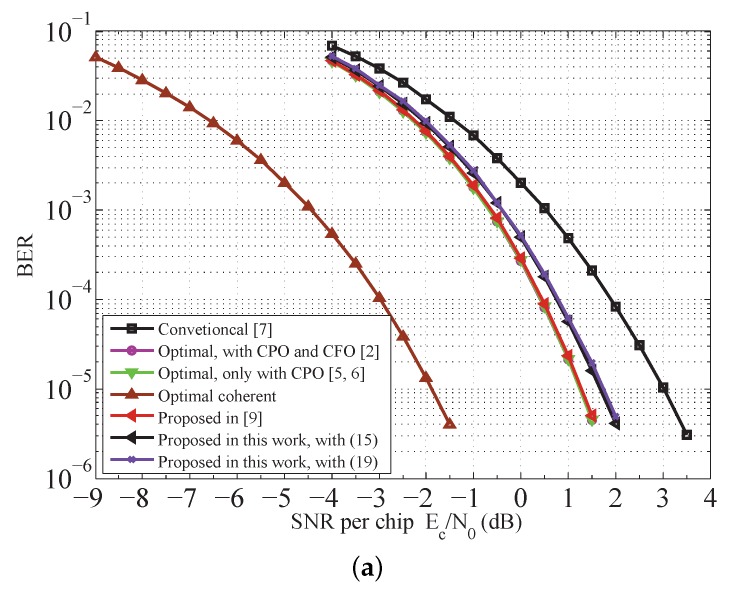

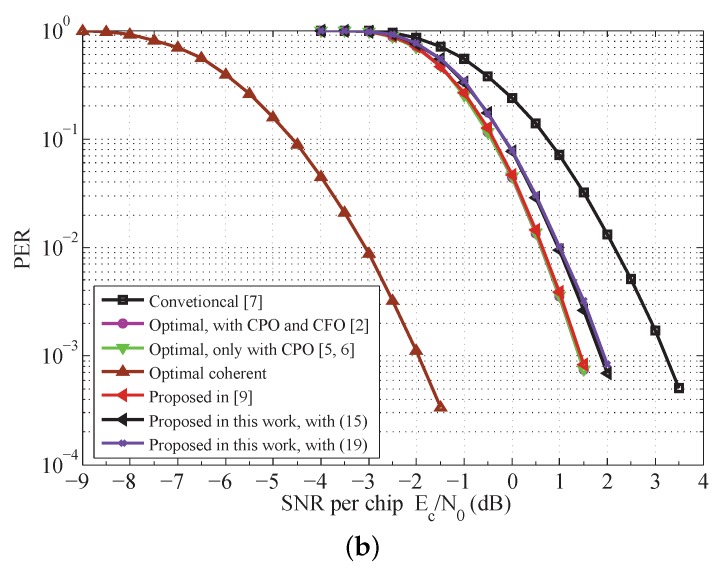
Performance comparison for various receivers. (**a**) BER performance of different receivers; (**b**) PER performance of different receivers.

The BER and PER performances of the proposed receiver versus others are shown in Figure 7. The optimal noncoherent receiver with random CPO is considered [6,7]. The ideal coherent (perfect carrier reference phase and no CFO) detection with SD decoding is also given for sake of comparison. Compared to the optimal noncoherent scheme, the CFOE can be compensated by a postcompensation processor to various degrees as shown in Figure 7. At PER of $1\times 10^{-3}$, our detection shceme achieves more than 1.3 dB gains over the method in [8]. The performance gap between the scheme in [10] and our detector is only about 0.2 dB; however, substantial reduction in complexity is achieved. Referring to Figure 7, only 1 dB is enough for our detector to meet specific requirements on the performance in [1]. In addition, our scheme is efficient at all SNR regions, although the approximation |Y|≈1 involved in Equation (19) is only valid at high SNRs. This follows from the fact that the BER and PER performances of the receiver with Equations (15) and (19) are almost the same as indicated in Figure 7.

**Figure 8:**
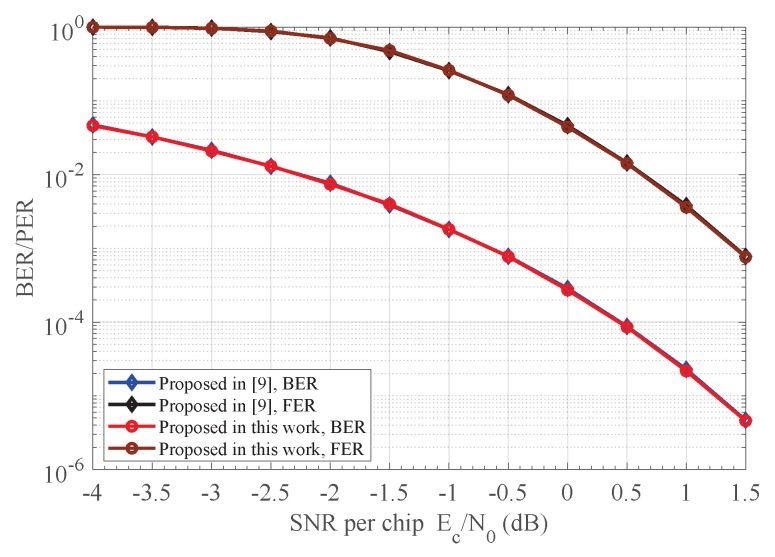
BER and PER comparison for the proposed receiver and the receiver in [10].

The BER and PER performances of the proposed receiver versus the receiver in [10] when the CFO was randomly set to range from −6.2 ppm (i.e., the CFOE φ is −0.288π) to 6.2 ppm (i.e., the CFOE φ is 0.288π) with triangular symmetric distribution are depicted in Figure 8. As shown in Figure 8, our proposed estimation method in Equation (19) compensates the frequency offset effect more efficiently than that in Equation (12), whereas substantial reduction in complexity is achieved at the same time. This follows from the fact that the absolute estimation error |e| of our proposed estimation method is now smaller than that of Equation (12) as indicated in Figure 5. Similar results were also achieved for the CFOE φ∈(−π,−0.712π)∪(0.712π,π), which are not given here.

### 7.3. Robustness of the Receiver

**Figure 9:**
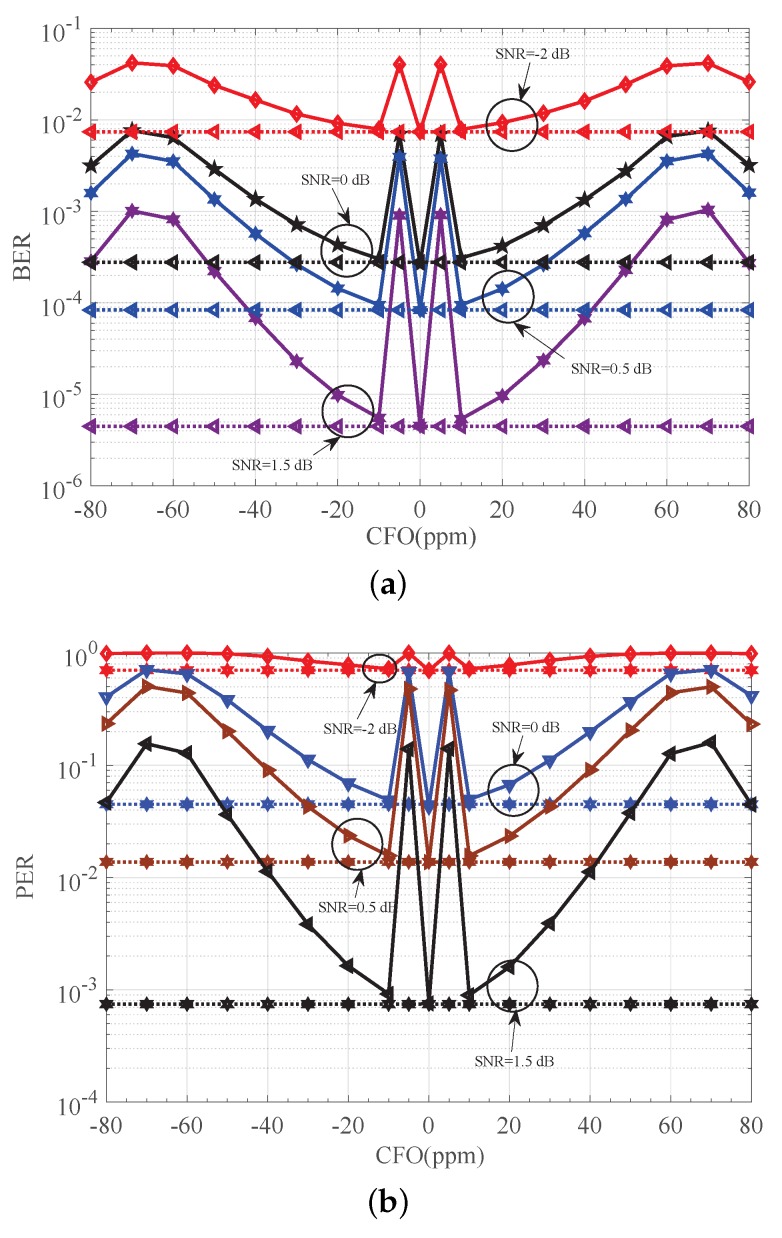
Detection performance of [8]. (**a**) BER performance of [8] versus CFO; (**b**) PER performance of [8] versus CFO. CFO: carrier frequency offset.

**Figure 10:**
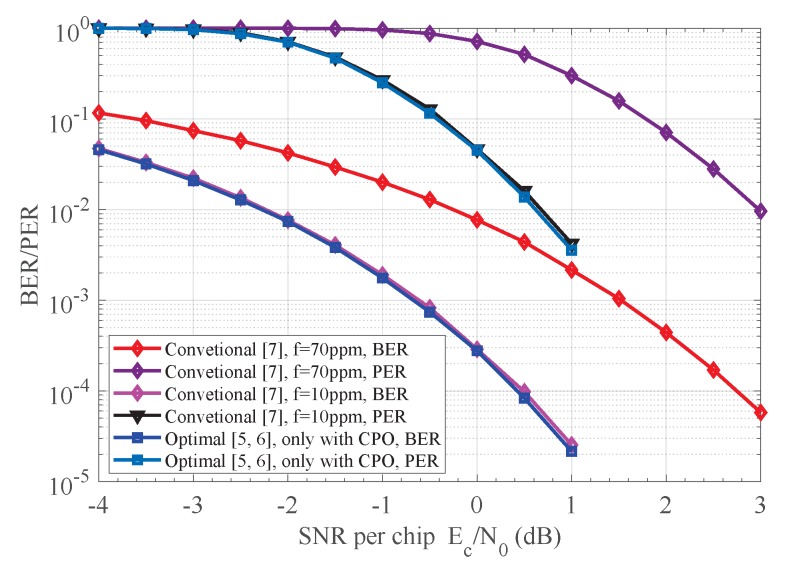
BER and PER performances of [8] versus two particular CFOs. CFO: carrier frequency offset.

The performance of the detector in [8] versus CFO is shown in Figure 9. In Figure 9, the horizontal line represents the detection performace when no CFO is considered, which gives us a benchmark for comparison. As indicated in Figure 9, good performance is achieved especially when *f* equals ±10 ppm for the detector in [8]. This result follows from the number of the gap between actual CFOE and its corresponding quantified value π/2 being small enough, i.e., only 0.0380π radians. However, when this number greatly increases to 0.2340π radians, i.e., f=±70 ppm, performance is degraded severely by large estimation error (e.g., more than 2 dB at PER of $1\times 10^{-2}$ as shown in Figure 10). The analysis result for the simulation in Figure 9 is described in detail in Table 3. The result for the absolute estimation error |e| is also presented in Table 3. Observe from Figure 9 and Table 3 that the smaller the absolute estimation error is, the better the detection performance. When |e| is close to its maximum value 0.25π (i.g., *f* equals ±5 ppm, ±60 ppm or ±70 ppm), and the receiver suffers from a serious performance penalty. Based on those observations, we see that the detector in [8] works well only for such CFOEs that are close enough to four values, i.e., 0,π/2,−π, and −π/2. Otherwise, a large estimation error will introduce unacceptable performance penalty.

**Figure 11:**
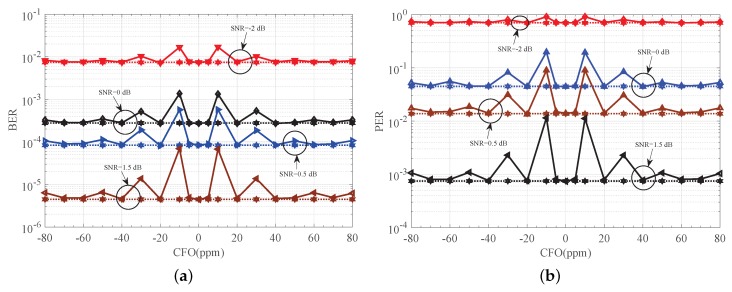
Detection performance of proposed receiver. (**a**) BER performance of proposed receiver versus CFO; (**b**) PER performance of proposed receiver versus CFO. CFO: carrier frequency offset.

The limitation in [8] is avoided to some extent in our detector as depicted in Figure 11. As indicated in Figure 11, good performance is achieved for most considered CFOs. This is because a simple additive adaptive offset term Im(Y) is introduced as shown in Equation (19), and the maximum value for the absolute estimation error |e| is reduced to π/2−1. In addition, only when |φ| is close enough to π/2 (i.g., the CFO *f* equals ±10 ppm), large degradation in performance is observed. This is because a relatively large approximation error in $\sin^{-1}\left(x\right)\approx x$ is now achieved as shown in Figure 5. However, note that no limitation on the CFOE range is required for direct application of the mathematical approximation $\sin^{-1}\left(x\right)\approx x$, for small *x*, in our estimator.

### 7.4. Performance under Dynamic Channel

**Figure 12:**
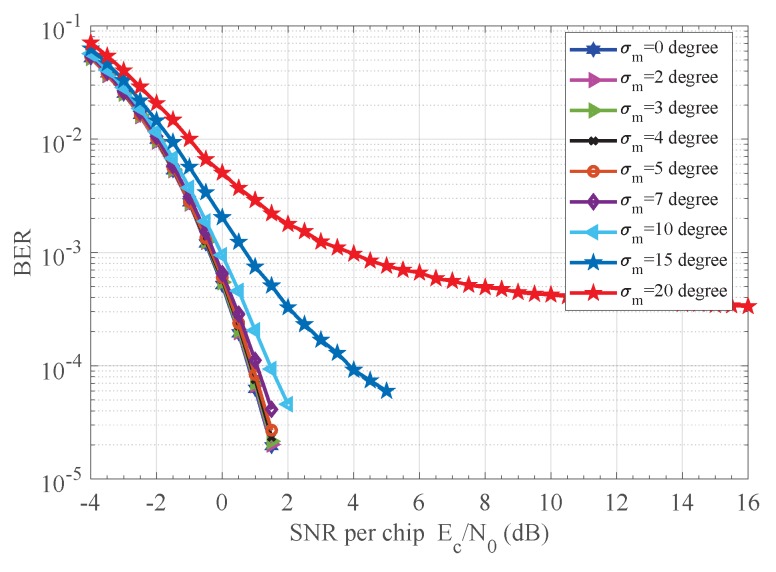
BER performance of proposed receiver versus dynamic CPO. CPO: carrier phase offset.

**Figure 13:**
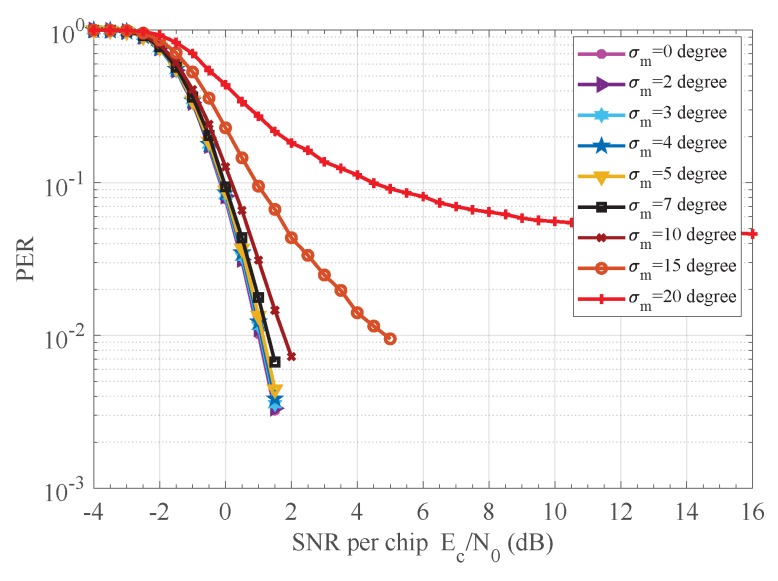
PER performance of proposed receiver versus dynamic CPO. CPO: carrier phase offset.

Finally, the behavior of the proposed receiver under dynamic channel conditions has been investigated. Our noncoherent receiver is robust to phase jitter as observed from Figure 12 and Figure 13. The phase θ of received chip sequence $\left\{r_{m,k}\right\}$, 1≤k≤K, is modeled as a Wiener process according to $\theta_{m+1}=\theta_{m}+\Delta_{m}$, where $\Delta_{m}$ are zero-mean independent Gaussian random variables with known variance $\sigma_{m}^{2}$ in each bit interval, and the initial phase $\theta_{1}$ is considered to be uniform distribution. A jitter standard deviation up to four degrees does not degrade significantly the receiver performance. Another feature of the curves in Figure 12 and Figure 13 is that they exhibit an irreducible error floor as SNR increases. Furthermore, the smaller the standard deviation is, the lower the error floor. The explanation is that the random phase increment $\Delta_{m}$ generates a phasor $e^{j\Delta_{m}}$ in the autocorrelator output A[m], which, in turn, produces decision errors even in the absence of noise. The smaller the standard deviation, the lesser the effect of the phasor $e^{j\Delta_{m}}$ on the autocorrelator output A[m].

## 8. Conclusions

We have presented a BSDCD scheme of BPSK signals for IEEE 802.15.4 WSNs. Then, a more meaningful and practical estimate scheme, i.e., an approximate ML estimator with only linear operation, is proposed for extracting a phase reference from preamble signal. Simulations suggest that our lower complexity in implementation does not sacrifice much in terms of detection performance, which can completely meet the requirements of the IEEE 802.15.4 standard. Therefore, it is the most attractive SDCD solution of choice for WSNs, especially when it used in consumer electronics.

It is important to note that our idea in [10] as well as in this work can be used for simplification of Equation (8) in [14], where arctan operation is indispensable to performing an initial CFO estimation process. Loosely speaking, these schemes can be further classified into the hard receiver. It makes use of only the estimates of the nuisance parameters as if they were the true values for the noncoherent detection. In the soft receiver, the postcompensation processor is required to compute posterioris or conditional probability density functions of the nuisance parameters embedded in the chip sequence [12]. Thus, the detection process can incorporate a statistical characterization of the nuisance parameters (instead of the estimated ones). The latter is, of course, nonimplementable in general.

Furthermore, unlike those who were researching ways to use BSDCD to combat the effect of chip-error propagation [9], we add that the theory developed here is easily extended to multiple differential coherent detection (MDCD). That is to say, the extension of the proposed scheme to account for multiple-symbols situation is straightforward. As a consequence, the performance gap between ideal coherent detection and BSDCD, indicated in Figure 7, can be narrowed. Not surprisingly, multiple unknown CFOEs after DD are necessary to be initially removed. Complexity reduction techniques may be developed based on the Viterbi algorithm [27], the fast algorithm in [28] and an algorithm based on subset search [29]. Of course, owing to the reduced PER that this augmented scheme yields, a much more reasonable trade-off can be achieved and is clearly desirable for both the battery-operated transmitter and receiver, which benefit from less energy that will be consumed by retransmissions between sensor nodes [30]. Finally, the complexity and energy efficiency can be deeply analyzed with the model given in [30]. These subjects will be reported on by the authors in a forthcoming paper.

## Figures and Tables

**Table 1:** Summary of the absolute error.

Region	Absolute Error |e|		
	Estimator in [8]	Estimator in [4]	Estimator in this work
[−π,−3π/4)	φ+π	(−π+tanφ)−φ	φ−(−π−sinφ)
[−3π/4,−π/2)	−φ−π/2	φ−(−π/2+cotφ)	φ−(−π−sinφ)
[−π/2,−π/4)	φ+π/2	(−π/2−cotφ)−φ	sinφ−φ
[−π/4,0)	−φ	φ−tanφ	sinφ−φ
[0,π/4)	φ	tanφ−φ	φ−sinφ
[π/4,π/2)	−φ+π/2	φ−(π/2−cotφ)	φ−sinφ
[π/2,3π/4)	φ−π/2	(π/2−cotφ)−φ	(π−sinφ)−φ
[3π/4,π)	−φ+π	φ−(π+tanφ)	(π−sinφ)−φ

**Table 2:** Parameters used in simulations. AWGN: additive white Gaussian noise; SNR: signal-to-noise ratio; PN: pseudorandom number; PPDU: physical layers protocol data unit; CPO: carrier phase offset.

Parameter	Detailed Description
Channel condition	Complex AWGN
Power of the complex noise	1/SNR
Detection Scheme	Bit-level noncoherent
Timing synchronisation	Perfect
Generator polynomial of PN code	$1+x+x^{4}$
Payload length of PPDU (bits)	160
Carrier frequency (MHz)	924
CPO (rads)	Uniform distribution in (−π, π) or Wiener process

**Table 3:** Robustness analysis for the detector in [8]. CFOE: carrier frequency offset effect.

CFO (ppm)	Performance Rank	Actual CFOE (Rads)	Estimated CFOE (Rads)	|e| (Rads)
5	Eighth	0.2310π	0	0.2310π
10	First	0.4620π	π/2	0.0380π
20	Second	0.9240π	−π	0.0768π
30	Third	−0.6275π	−π/2	0.1275π
40	Fourth	−0.1520π	0	0.1520π
50	Fifth	0.3100π	π/2	0.1900π
60	Seventh	0.7720π	−π	0.2280π
70	Ninth	−0.7660π	−π	0.2340π
80	Sixth	−0.3040π	−π/2	0.1960π
